# Does Environmental Regulation Promote Environmental Innovation? An Empirical Study of Cities in China

**DOI:** 10.3390/ijerph19010139

**Published:** 2021-12-23

**Authors:** Dezhong Duan, Qifan Xia

**Affiliations:** Institute for Global Innovation and Development, East China Normal University, Shanghai 200062, China; 51183902025@stu.ecnu.edu.cn

**Keywords:** environmental regulation, environmental innovation, environmental patent, spatial Durbin model, China cities

## Abstract

Promoting environmental innovation through environmental regulation is a key measure for cities to reduce environmental pressure; however, the role of environmental regulation in environmental innovation is controversial. This study used the number of environmental patent applications to measure urban environmental innovation and analyzed the role of urban environmental regulation on urban environmental innovation with the help of the spatial Durbin model (SDM). The results showed that: (1) From 2007 to 2017, the number of environmental patent applications in China has grown rapidly, and technologies related to buildings dominated the development of China’s environmental innovation. (2) Although the number of cities participating in environmental innovation was increasing, China’s environmental innovation activities were highly concentrated in a few cities (Beijing, Shenzhen, and Shanghai), showing significant spatial correlation and spatial agglomeration characteristics. (3) Urban environmental regulation had a positive U-shaped relationship with urban environmental innovation capability, which was consistent with what the Porter hypothesis advocates.

## 1. Introduction

The urban environment is a major contributor to climate change, mainly due to its high dependence on natural resources [1]; therefore, cities can be key players in combating climate change by addressing environmental sustainability actions (e.g., formulating pollution discharge standards, collecting pollution taxes, granting emission reduction subsidies, issuing pollution permits, etc.) to encourage enterprises to carry out environmental innovation in order to reduce emissions [2]. China has ushered in rapid urbanization for nearly 40 years under the “competition for growth” economic development mode since reform and opening-up; however, it is also facing increasingly severe urban environment pressures [3]. It is undeniable that the Chinese government attaches increasing importance to environmental protection, and increasingly strict environmental regulations are constantly being implemented in China [4].

The realization of strong decoupling between urban economic growth and environmental degradation crucially depends on technological improvements, which is environmental innovation. Environmental innovation is a core topic in research fields, such as environmental economics, innovation economics, and innovation management, emphasizing innovative behavior based on environmental protection or reducing environmental damage. The academic community has carried out a great deal of research on environmental innovation, including the assessment of environmental innovation ability, the determinants of environmental innovation, the economic and environmental effects of environmental innovation, environmental innovation policy design, etc. Since environmental innovation puts more emphasis on production and output [5,6], research on environmental innovation at the enterprise level has become the mainstream [7]. Similarly, environmental innovation is generally considered to be a response of enterprises to government environmental regulations. However, the academic circle has not yet formed a conclusion regarding the relationship between environmental regulation and environmental innovation, and rather has formed a binary opposition between two theories, namely the “Porter hypothesis” [8] and the “repression hypothesis” [9]. In addition, many scholars hold a wait-and-see attitude, indicating that the relationship between the two needs more verification [10].

Recently, with the promotion of regional innovation system theory [11], the relationship between environmental regulation and environmental innovation (green innovation efficiency, green development efficiency) has become a hot topic in environmental economic geography, regional economics, urban economics, innovation geography, and other fields [12,13]. Due to the large regional differences and the local competition brought by decentralization, China is the best-case field for this hot research topic. Many studies have revealed the positive correlation between environmental regulations and environmental innovation at the regional level in China [14]; however, there are some studies that have found a non-linear relationship between China’s urban environmental regulation and urban environmental innovation, showing a U-shaped pattern. That is, the effect of environmental regulation on environmental innovation shows the characteristics of restraint in the short term and promotion in the long term [13]. In addition, some scholars believe that the impact of China’s environmental regulation on environmental innovations presents significant spatial heterogeneity; that is, the relationship between the two in eastern China is significantly positive, while the relationship between the two in the central and western regions needs more discussion [15].

Looking at the existing regional or city-level related research, it is not difficult to find that the relationship between environmental regulation and environmental innovation still needs to be tested with more empirical research. One of the main reasons for the differences in existing research results is that scholars have used different research methods in measuring regional or urban environmental innovation capabilities. Some use the input–output analysis method to measure the efficiency of regional environmental innovation [14], some use the number of environmental patent applications (not completely covering all patent categories) to measure the environmental innovation capability [15], and others use alternative indicators to measure regional environmental innovation capability [16].

The purpose of this paper is to explore the relations between environmental regulation and environmental innovation in China from a city-level perspective. The main contributions are twofold. First, this paper has enriched the literature that uses patent data to measure urban environmental innovation. Based on the contribution of Haščič and Migotto [17], we analyzed the developments of urban environmental innovation in China from 2007 to 2017 by identifying the environment-related patents. Second, this paper has also enriched the literature on the relationships between environmental regulation and environmental innovation. Based on the spatial econometric model, we explored the relationship between urban environmental regulation and environmental innovation in China.

The remainder of the article is organized as follows. Section 2 provides a short literature review of the current research on the relationship between environmental regulations and environmental innovation. Section 3 introduces the data and methodology used in this paper. Section 4 presents the empirical results, and Section 5 discusses our findings and concludes this paper.

## 2. Theoretical Background

Exploring the relations between environmental regulation and environmental innovation is the core issue of environmental economics and other related disciplines. Environmental economics regards environmental regulation as a policy tool for internalizing the external costs of enterprises, focusing on the impact of environmental regulation on enterprises and the impact of different policy tools on enterprise innovation behavior. However, what kind of impact will environmental regulation have on environmental innovation? This has always been a controversial and interesting topic. The mainstream view agrees that environmental regulation can induce innovation and bring about innovation compensation and competitive advantage; this is the “Porter Hypothesis” [9], which states that market-oriented policy instruments (such as pollution taxes, emission reduction subsidies, and pollution permits) can stimulate enterprises to carry out environmental innovation more than command-and-control regulations (such as pollution standards, emission quotas, etc.) [18].

However, there are also views that strict environmental regulations may have a negative impact on enterprises, and will not only increase the production cost of the enterprise and the survival risk of the enterprise, but also bring about the transfer of pollutants. For example, some companies experienced a severe decline in financial performance after implementing environmental innovation and did not achieve Porter’s “win-win” situation [19]. Some studies also found that environmental regulation can lead to the decline of enterprise productivity, even producing negative spillover effects on the national economy. Barbera and McConnell [20] found that the main reason for the decline in the performance of steel, nonferrous metals, paper, chemical, and non-metallic mineral products industries in the United States was the increase in pollution control investment caused by environmental regulation. Moreover, environmental regulations will have different effects on enterprises of different sizes and types. Numerous studies have shown that environmental innovation practices vary greatly between companies. Large companies, especially multinational companies, are pioneers and leaders in environmental innovation, while Small and Medium Enterprises (SMEs) have shown more uncertainty in environmental innovation [21,22]. In addition, according to the research of Ouyang et al. (2020) concerning China, environmental supervision is not conducive to technological innovation of state-owned enterprises due to the high cost of energy conservation and emission reduction, and industries with higher market competition and human capital investment tend to have stronger environmental innovation capabilities. It was also found that the scale of the regional economy also has a significant impact on the regional environmental innovation capacity [3,23,24].

Contrary to the above two opposing views, some scholars hold that the impact of environmental regulation on green technology innovation is uncertain. For example, Mody [25] found that there was no obvious correlation between the increased emission reduction expenditure of environmental regulation and environmental innovation. Jaffe and Palmer’s research [26] on US manufacturing showed that the impact of environmental regulation on environmental innovation is not significant. In summary, the relations between environmental regulation and environmental innovation have aroused controversy in academia. Research conclusions are often different due to differences in research cases (industry differences, scale differences, type differences, etc.) and measurement methods (measures of environmental regulation and measures of environmental innovation). Environmental innovation is widely believed to be the strategic adjustment of enterprise development in response to environmental regulation to gain competitive advantages; thus, the relations between environmental regulation and environmental innovation at the enterprise level have always been the focus of attention [27], while research on the relationship between environmental regulation and environmental innovation at the city level is still rare.

Based on the perspective of urban space in China, this study first measured the intensity of urban environmental regulation and urban environmental innovation capability in China, and then used a spatial econometric model to explore the relationship between environmental regulation and urban environmental innovation ability, to make up for the spatial factors that have not been fully considered in previous studies.

## 3. Data and Methods

### 3.1. Measuring Environmental Regulation

Measuring the intensity of environmental regulation is the first step to test the relationship between environmental regulation and environmental innovation. Existing literature has launched an active attempt to quantify environmental regulations, which can be roughly divided into four categories.

The first is to use environmental governance expenditures to measure the intensity of environmental regulations. Related indicators include the government investment in pollution control and other incentive policies [28], pollutant emission reduction expenditure, and other regulatory measures [29]. The second is measurement of the emission level of pollutants or treatment level, such as the scale of pollutant emissions, pollutant treatment rate, domestic sewage treatment rate, etc. The third measurement method is based on the input–output analysis framework to build an environmental regulation intensity evaluation system. For example, Sauter [30] believed that environmental regulation is an input–output process, and the measurement of environmental regulation should include three dimensions: input, process, and result. The last method is to use alternative indicators to measure environmental regulation [31]. To avoid the complexity of the measurement of environmental regulation indicators, some studies have used alternative indicators to express the degree of environmental regulation, such as the lead content in gasoline [32] and the income level of residents [33].

Comparing the four methods mentioned above, it seems that the comprehensive construction of an environmental regulation evaluation system is a very reasonable description method; however, due to the limitations of data availability and index screening, it is difficult to apply to city-level research. Moreover, it is controversial to use alternative indicators to measure environmental regulation. For example, the income of residents in developed countries may reflect the high requirements for environmental protection, while in many developing countries, due to the limitation of the development stage, most areas may be at the left end of the environmental Kuznets curve; that is, a higher per capita GDP may correspond to a lower intensity of environmental regulation. Placing the research in the context of China, when the lower limit of the research scale is cities rather than provinces, the data for pollutant emission reduction expenditure investment is almost impossible to obtain; however, the pollutant emission level has official statistics. 

According to past experiences [4], this paper constructed a comprehensive index of environmental regulation intensity in China city systems based on three indicators: the comprehensive utilization rate of industrial solid waste, domestic sewage treatment rate, and domestic waste harmless treatment rate. The relevant data are from the China City Statistical Yearbook and the specific methods are as follows: firstly, the extreme value method was used to standardize the treatment rate of the three pollutants (Equation (1)); second, considering the differences in the discharge of pollutants in each city, if a city had a large discharge of pollutants, the same treatment rate for such pollutants may mean stricter environmental regulations, so a greater weight was assigned; third, the weighted average of the treatment rates of three pollutants in a city was taken as the intensity of the city’s environmental regulations (Equation (2)).

(1) $$S\_Pr_{i,t}^{g}=\left[Pr_{i,t}^{g}-\min\left(Pr_{t}^{g}\right)\right]/\left[\max\left(Pr_{t}^{g}\right)-\min\left(Pr_{t}^{g}\right)\right]$$

In the above formula, $S\_Pr_{i,t}^{g}$ and $Pr_{i,t}^{g}$ are the standard and original value of the treatment rate of pollutant *g* in city *i* at time *t*, respectively. $\max\left(Pr_{t}^{g}\right)$ and $\min\left(Pr_{t}^{g}\right)$ are the maximum value and minimum value, respectively, of the original values of the treatment rates of pollutant *g* in China as a whole.

(2) $$Environ\_Regu_{i,t}=\left[\sum_{g=1}^{3}\left(\frac{P_{i,t}^{g}}{P_{t}^{g}}\right)*S\_Pr_{i,t}^{g}\right]/3$$

In Equation (2), $Environ\_Regu_{i,t}$ is the comprehensive index of environmental regulation intensity in city *i* at time *t*. $Pr_{t}^{g}$ is the emission of pollutant *g* in China as a whole.

### 3.2. Measuring Environmental Innovation

Given its multi and transdisciplinary features, there are many concepts related to environmental innovation, such as green innovation, eco innovation, and sustainable innovation [34]. Due to this unclear definition, these concepts are usually interchangeable [35]. However, in terms of measurement, studies to date have employed different perspectives and methods. Existing environmental innovation measurements also can be classified into four categories. 

The first is the overall measurement of environmental innovation by constructing an evaluation system covering as many indicators as possible [36,37]. The second is to focus on environmental innovation input, usually measured by environmental R&D investment [38]. The third is to focus on environmental innovation output, usually measured by environmental patents [17,39]. Due to their quantifiable and available features, patents, especially those related to the environment, are widely used to measure environmental innovation [40,41]. The fourth is to focus on the process of environmental innovation, usually using environmental innovation efficiency as a proxy for environmental innovation [42,43,44]. Existing research has completed the measurement of the environmental innovation efficiency of some enterprises (large enterprises, SMEs, or specific enterprises), cities, regions, and countries [42].

Certainly, there are other methods for measuring environmental innovation; however, they are relatively rare and impractical. For example, determining green products based on industry and commodity classification would first require identification of the industry or commodity classes that represent environmental. In this paper, we used environmental patents to measure China’s environmental innovation at the city level due to their higher availability and wider coverage when compared to environmental R&D investment, green products, and other indicators.

Through referencing the identification strategies of environment-related technologies (patents) proposed by Haščič and Migotto [17], this study obtained environmental patent application data from the China Wanfang patent database (http://g.wanfangdata.com.cn/index.html, accessed on 1 December 2021). The environment-related technologies (see Table A1 in Appendix A) in this paper include energy technology (climate change mitigation technologies, related energy generation, transmission of distribution etc.), greenhouse gas treatment technology (capture, storage, sequestration, or disposal of greenhouse gases), transportation technology (climate change mitigation technologies related to transportation), building technology (climate change mitigation technologies related to buildings), environmental management technology (technologies of air pollution abatement, water pollution abatement, waste management, soil remediation, and environmental monitoring), and water-related adaptation technology (technologies of water conservation and availability). We assigned these environmental patents to cities in China based on the address of the patent applicant, including municipalities directly under the central government (e.g., Beijing, Shanghai, Tianjin, and Chongqing), prefecture level cities (e.g., Hangzhou, Suzhou, Guangzhou, Shenzhen, Nanjing, etc.), autonomous prefectures (e.g., Dali Bai Autonomous Prefecture, Enshi Tujia and Miao Autonomous Prefecture, Chuxiong Yi Autonomous Prefecture, etc.), prefecture regions (e.g., Altay, Ali, Aksu, etc.), leagues (e.g., Xilingol League, Alashan League, and Xingan League) and counties directly under the jurisdiction of provinces (e.g., Xiantao, Qianjiang, Tianmen, etc.).

Table 1 shows the development trend of environmental innovation in China in the past decade. From 2007 to 2017, environment-related patent applications increased from 87,691 to 307,929, with an average annual growth rate of 13.4%. In addition to being slightly lower than the field of energy in 2010, technologies related to buildings have always ranked first, increasing from 35,850 in 2007 to 105,681 in 2017.

Table 2 shows the proportion of environmental patents in Chinese patent applications. Although the number of environmental patent applications has increased rapidly, its proportion in all patent applications has shown a significant decline. At the urban scale, the proportion of environmental patent applications in each city was also much lower than that of non-environmental patents. This seems to indicate that environmental innovation has a higher production cost and a higher production threshold.

### 3.3. Research Methodology

#### 3.3.1. Variables 

The interpreted variable in our study was urban environmental innovation capability (EIC). Urban EIC was measured by the environmental patent applications.

The core explanatory variable was urban environmental regulation (ER). Referring to the comprehensive index construction methods of Peng [4], this study selected three indicators, namely the comprehensive utilization rate of industrial solid waste, domestic sewage treatment rate, and domestic waste harmless treatment rate.

Innovation economics believes that, as an innovative activity that emphasizes environmental protection or reduces environmental damage [34,35], the market drivers of general innovation behavior (population, foreign direct investment (FDI), economic development level, urban construction scale, etc.) and technology drivers (investment, human capital, etc.) are also applicable and effective for explaining the determinants of environmental innovation [16,40,45]. For example, the relationship between FDI and environmental innovation has been widely explored in the fields of innovation economics and innovation management [46,47,48]. With the in-depth exploration of evolutionary economic and grounded theory, scholars have found that innovation willingness and innovation attitude have an important role in promoting environmental innovation, and environmental innovation is highly dependent on its own development mode, growth path, and industry environment. Meanwhile, environmental innovation is a means for enterprises (especially manufacturing) to respond to environmental regulations and participate in market competition. If a city’s industrial structure is dominated by industrial manufacturing, it would bear more responsibility for environmental protection and pollution reduction, and the city will also introduce more environmental policies to stimulate enterprises to carry out environmental innovation [3,13]. Besides, in China, different cities have different administrative levels. Cities with different administrative levels have certain differences in the level of educational resources, investment in innovation resources, and convenience of patent application or transfer. 

Therefore, based on the existing innovation and environmental innovation literature, we added eight control variables: urban size (U-S), urban FDI (U-FDI), urban economic development level (U-EDL), urban technological innovation capability (U-TIC), urban construction scale (U-CS), urban initial environmental innovation capacity (U-IEIC), urban industrial structure (U-IS), and urban administrative level (U-AL). In terms of the measurement of these indicators, this study used the urban population to characterize the U-S, used the amount of foreign capital used in that year to characterize the U-FDI, used the urban per capita GDP to measure the U-EDL, used the proportion of secondary industry to measure the U-IS, and used the number of environmental patents of the city in 1990 to characterize U-IEIC (the China National Intellectual Property Administration (CNIPA) began collecting patent data in 1985, and considering the incompleteness of the data in the first few years, the data of 1990 was adopted). Regarding U-AL, we constructed a dummy variable, which was 1 if the city is a provincial capital, and 0 otherwise. Unless otherwise noted, data on control variables were obtained from China City Statistical Yearbook.

As discussed above, Table 3 lists and describes the interpreted, core explanatory, and other control variables.

#### 3.3.2. Spatial Correlation Analysis 

To explore the spatial correlation of environmental innovation in China, this study used the global Moran’s I index to conduct a spatial statistical analysis of environmental innovation in China city systems [12]. The definition of the global Moran’s I index is:

(3) $${\mathrm{Moran}}^{’}s\;I=\frac{n\sum_{i=1}^{n}\sum_{j=1,i\neq j}^{n}W_{ij}\left(X_{i}-\bar{X}\right)\left(X_{j}-\bar{X}\right)}{\sigma^{2}\sum_{i=1}^{n}\sum_{j=1,i\neq j}^{n}W_{ij}},\;\bar{X}=\frac{1}{n}\overset{n}{\underset{i=1}{\sum }}X_{i},\;\sigma^{2}=\frac{1}{n}\overset{n}{\underset{i=1}{\sum }}{\left(X_{i}-\bar{X}\right)}^{2},\;W_{ij}=1/d_{\mathrm{ij}}$$

where $X_{i}$ and $X_{j}$ represent the *EIC* in city *i* and city *j*, respectively. $W_{ij}$ is the spatial weighted matrix, *n* is the number of cities, and *d_ij_* is the distance between city *i* and city *j*. The range of values of the global Moran’s I index is [−1, 1].

Table 4 shows the global Moran’s I index results. The index of urban *EIC* in each period was positive at the 1% level, and the Moran’s I of the six types of environment-related technologies all showed a rising trend that was statistically significant at *p* < 0.01, indicating a significantly positive spatial autocorrelation in the urban *EIC* of the 349 cities in China from 2007 to 2017.

#### 3.3.3. Random Effects Model

For the coldiag2 test, the condition number using scaled variables was 13.02; thus, the variables passed the collinearity test [49]. To ensure the accuracy and credibility of the estimated results of the F test, the fixed-effect model is considered to be significantly better than mixed regression. Further, considering the addition of dummy variables and according to the least squares dummy variables (LSDV) method, a Hausman test was used to determine the use of random effects model.

#### 3.3.4. Spatial Regression Model

Due to the significant spatial correlation of environmental innovation with cities in China, a spatial panel regression model was considered for the detection of the determinants. By comparing the spatial lag model (SLM), the spatial error model (SEM), and the spatial Durbin model (SDM) based on Stata 12.0 analysis, it was found that the goodness of fit and credibility of the SDM (*R*^2^ = 0.863) was the highest among the three models, and the Hausman test results showed that the SDM random effects passed the robustness test. Therefore, this paper selected the SDM with random effects to analyze the determinants of environmental innovation of cities in China:

(4) $$\begin{array}{ll} \mathrm{Ln}EIC_{i,t}=\alpha_{i}+ & \beta_{1}\mathrm{Ln}ER_{i,t}+\beta_{2}\mathrm{Ln}U-S_{i,t}+\beta_{3}\mathrm{Ln}U_{FDI}{}_{i,t}+\beta_{4}\mathrm{Ln}U-TIC_{i,t}+\beta_{5}\mathrm{Ln}U-IEIC_{i,t}+\beta_{6}\mathrm{Ln}U-CS_{i,t}\\ & +\beta_{7}\mathrm{Ln}U-EDL_{i,t}+\beta_{8}\mathrm{Ln}U-IS_{i,t}+\beta_{9}\mathrm{Ln}U-AL_{i,t}+\lambda \overset{11}{\underset{k=1}{\sum }}w_{ij}\mathrm{Ln}EIC_{j,t}+\theta_{1}\overset{11}{\underset{j=1}{\sum }}w_{ij}\mathrm{Ln}ER_{j,t}\\ & +\theta_{2}\overset{11}{\underset{j=1}{\sum }}w_{ij}\mathrm{Ln}U-S_{j,t}+\theta_{3}\overset{11}{\underset{j=1}{\sum }}w_{ij}\mathrm{Ln}U-FDI_{j,t}+\theta_{4}\overset{11}{\underset{j=1}{\sum }}w_{ij}\mathrm{Ln}U-TIOC_{j,t}\\ & +\theta_{5}\overset{11}{\underset{j=1}{\sum }}w_{ij}\mathrm{Ln}U-IEIC_{j,t}+\theta_{6}\overset{11}{\underset{j=1}{\sum }}w_{ij}\mathrm{Ln}U-CS_{j,t}+\theta_{7}\overset{11}{\underset{j=1}{\sum }}w_{ij}\mathrm{Ln}U-EDL_{j,t}\\ & +\theta_{8}\overset{11}{\underset{j=1}{\sum }}w_{ij}\mathrm{Ln}U-IS_{j,t}+\theta_{9}\overset{11}{\underset{j=1}{\sum }}w_{ij}\mathrm{Ln}U-AL_{j,t} \end{array}$$

where *i* indexes the city, and *t* indexes time.

## 4. Empirical Results

### 4.1. Environmental Innovation of Cities in China

From 2007 to 2017, the number of cities participating in environmental innovation in China increased from 330 in 2007 to 338 in 2017, among which the number of cities engaging in innovation around building technology was always the largest, while the number of cities engaging in innovation around greenhouse gas technology was always the smallest. Spatially, China’s environmental innovation activities showed significant spatial heterogeneity, highly concentrated in a few cities (Table 5).

Specifically, in 2007, the top 10 cities in environmental innovation accounted for 48.1% of the environment-related patent applications in China. There were four cities with more than 5000 environment-related patents, namely Shanghai (9003), Beijing (8073), Guangzhou (6689), and Shenzhen (6174). Shanghai also ranked first in patent applications in the field of greenhouse gases, building, water adaptation, and transportation technology, while Beijing ranked first in the field of environmental management and energy technology. In different technical fields, most of the cities with outstanding performance were in eastern China. Cities in central and western China were generally backward in environmental innovation. In 2012, the proportion of the top 10 cities in environmental patents fell to 43.3%. Beijing not only surpassed Shanghai in terms of total volume, but also ranked first in all six technical fields. Chengdu and Xi-An in the central and western regions ranked sixth and seventh with 3852 and 3822 patent applications, respectively. Similarly, in different technical fields, cities with outstanding performance were mostly located in east China. Environmental innovation in central and western China was still generally backward. By 2017, Beijing continued to rank first with 26,224 patent applications and Shenzhen ranked second with 18,997 patents. Shanghai, Guangzhou, and Suzhou ranked third, fourth, and fifth with 14,801, 11,800, and 10,659 patent applications, respectively. Foshan ranked tenth with 7059 environmental patent applications. Its environmental patent applications mainly came from the field of water-related adaptation, accounting for 57.7% of the total. This is mainly because the Midea Group, which is headquartered in Foshan, applied for more than 2000 patents in the field of water-related adaptation technology in 2017. In the six categories of environment-related technologies, Beijing still ranked first in environmental management, energy, greenhouse gases, and transportation technologies, while Shenzhen ranked first in the technologies of building and water-related adaptation.

### 4.2. Regression Results

Our initial data covered all cities at the prefecture level and above in China; however, due to the limitation of variable data, we finally selected 274 cities to enter the regression model. Table 6 shows the variable descriptive statistics, interpreted variables including urban EIC (total number of environmental patents), and the innovation ability of the cities in six types of environmental technology (the number of patent applications in each technology). Table 7 shows the descriptive statistics of the variables in three major regions of China. There were 114 cities in the eastern region, 108 in the central region, and 52 in the western region. 

Table 8 shows the regression results of the random effects model. The quadratic coefficients of environmental regulation were greater than 0, and the coefficients of the primary term were less than 0, which indicated that the impact of environmental regulation on the urban environmental innovation capability was a positive U-shaped curve that first declined and then rose. In other words, our research showed that urban environmental regulation had a restraining effect on urban environmental innovation in the short term [8]; however, they presented a positive correlation in the long term [12]. To avoid the interference of regional differences and improve the robustness and accuracy of the findings, we performed the same analysis on the sample data from three regions in China. Table 9 shows the regression results of different regions of China. We obtained the same results from the analysis, which showed that at the city level, the U-shaped relationship between China’s environmental regulation and environmental innovation has a certain degree of stability.

Furthermore, we continued to investigate whether the negative effect of environmental regulation on environmental innovation had spatial spillover effects or not. Table 10 reports the results of the SDM. Since the SDM contains the spatial lag terms of both the explanatory variables and the explained variables, the partial differential method was adopted to decompose the spillover effects of the SDM into direct effect, indirect effect, and total effect. Among them, the direct effect reflects the impact on the city’s environmental innovation, that is, the local effect. The indirect effect reflects the impact on the environmental innovation of the surrounding cities, that is, the spillover effect. The total effect is equal to the sum of the direct effect and the indirect effect. The R^2^ was 0.852 and the spatial rho was significant at a significance level of 1%, with a coefficient of 0.213, indicating that environmental innovation in China city systems has a significant positive spatial spillover effect. Regarding the relationship between environmental regulation and environmental innovation, the regression results of the SDM were consistent with the results of the random effects model. That is, the effect of environmental regulation on environmental innovation was first to inhibit and then to promote.

Through the above regression results, we verified some existing findings. Firstly, there was a significant positive correlation between urban size and urban environmental innovation capability, which is consistent with the existing studies on the relationship between enterprise environmental innovation and enterprise size [21,22], and the relationship between regional environmental innovation and regional size [3,23,24]. Secondly, the higher the level of urban economic development was, the stronger the capability of urban environmental innovation was. This is also consistent with the existing research results of environmental economics and innovation economics. Thirdly, urban FDI was not only positively correlated with the urban environmental innovation capability, but also showed positive local effects and local spillover effects. This is consistent with existing research results [38,45,47,48]. A large amount of foreign capital investment promotes the improvement of local environmental innovation ability, and also forms a strong trickle-down effect to the surrounding areas, promoting the environmental innovation of surrounding cities. Fourthly, the urban initial environmental innovation capability was obviously conducive to the urban environmental innovation capability. The direct effect of the urban initial environmental innovation capability was 18.288, and the indirect effect was −22.658, both of which were significant at the 1% significance level, indicating that urban environmental innovation had strong path dependence characteristics, while it also reflected that the cities with good performance in environmental innovation in the early stage would form a siphon effect, thus restraining the environmental innovation of surrounding cities.

In addition, we also found some very interesting conclusions about the control variables. Firstly, the industrial structure dominated by the secondary industry not only did not promote urban environmental innovation, but also played an obvious inhibitory effect. This finding is clearly different from the existing related research [3,12]. To verify the correctness of this result, we replaced this variable with the proportion of tertiary industry and performed a regression analysis again and found that the industrial structure dominated by the tertiary industry had a significant role in promoting the environmental innovation of the city itself and its surrounding cities. The reason for this result may be that the traditional manufacturing industry is still the pillar industry in China’s cities dominated by secondary industry, and patent applications are mostly completed by the tertiary industry, represented by the information and communication industry, real estate industry, and scientific research. Secondly, the scale of urban construction measured by the scale of urban fixed asset investment had a significant negative effect on urban environmental innovation capability. As an important driving force of economic growth, China’s urban fixed asset investment is growing rapidly. However, while stimulating economic growth, it has also increased energy consumption and environmental pollution. Many studies have found that there is a significant positive correlation between urban fixed asset investment and urban environmental pollution emissions in China [50]. Thirdly, there was a negative correlation between the urban administrative level and urban environmental innovation capability. The reason for this may be that some non-capital cities have performed very well in environmental innovation, such as Suzhou, Ningbo, Shenzhen, and Foshan. However, this negative correlation did not pass the significance test, which also showed that the relationship between the two needs more verification.

## 5. Conclusions

Exploring the relationship between environmental regulation and environmental innovation is the core topic of environmental economics, innovation economics, and other research fields, and it is also one of the emerging topics in the field of environmental economic geography in recent years. This study used the number of environmental patent applications to measure urban environmental innovation, and analyzed the role of urban environmental regulation on urban environmental innovation, from which we summarize the following key findings.

Firstly, the number of environmental patents in China has grown rapidly, from 87,691 in 2007 to 307,929 in 2017. From a technical perspective, technologies related to buildings have always dominated the development of environmental innovation of cities in China, while technologies in the field of greenhouse gases and water adaptation were quite unpopular throughout China. China’s urban environmental innovation showed a significant spatial correlation. The Moran’s I index of both the whole (urban EIC) and the six technical fields were significant at the 1% level and greater than 0. Additionally, in the time sequence, the values of Moran’s index were increasing, which indicated that the spatial correlation was strengthened with the passage of time. 

Secondly, the number of cities participating in environmental innovation in China increased from 330 in 2007 to 338 in 2017, among which the number of cities engaging in innovation around building technology was always the largest, while the number of cities engaging in innovation around the greenhouse gas technology was always the smallest. Spatially, China’s environmental innovation activities showed significant spatial heterogeneity, highly concentrated in a few cities [43]. From 2007 to 2017, Beijing not only surpassed Shanghai in the total number of environmental patent applications, but also ranked first in the fields of environmental management, energy, greenhouse gas treatment, and transportation technology. Shenzhen also surpassed Shanghai in the total number of environmental patent applications, ranking second in China. At the same time, Shenzhen had the largest number of environmental patent applications in the field of building and water-related adaptation in China.

Thirdly, both the random effects model and the SDM model showed that there was a U-shaped relationship between China’s urban environmental regulation and urban environmental innovation, which was not only consistent with what the Porter hypothesis advocates [9,12], but is also consistent with existing research on the relationship between green innovation and urban green development [51]. Moreover, the regression results of different technical fields and different regions verified this result. These results showed that environmental regulations will first restrict environmental innovation due to increased production costs. After a period of adaptation, environmental regulations will induce environmental innovation. We also found some interesting results in the control variables. Urban size, urban economic development level, and urban FDI all played positive roles in promoting urban environmental innovation, while the urban fixed asset investment scale and industrial structure dominated by the secondary industry significantly inhibited urban environmental innovation. In addition, we also found that urban environmental innovation had significant path dependence characteristics. 

## 6. Discussion

The discussion of the relationship between environmental regulation and environmental innovation in this article gives us many policy implications. Firstly, local governments should adhere to the innovation-driven development strategy, increasing R&D investment to promote the continuous growth of urban technological innovation capability. Secondly, local governments should continue to promote the transformation and upgrading of the urban industrial structure, especially increasing the supports for the producer service industry. Thirdly, local governments should increase the introduction of foreign capital to promote the upgrading of local production and management methods. Fourthly, local governments should conduct environmental assessments on the increasing urban fixed asset investment under the background of rapid urbanization. In addition, increasing investment in fixed assets will also reduce other financial expenditures. Fifthly, in view of the U-shaped relationship between environmental regulation and environmental innovation, local governments should adopt strategies that adapt to time and local conditions in environmental regulation.

We noticed that enterprises are becoming increasingly important as the largest actors in China’s environmental innovation. Based on applicant information for environmental patents, we identified environmental innovation actors in four categories, namely universities and scientific research institutions, enterprises, individuals, and others. We found that the proportion of enterprises increased rapidly from 39.7% in 2007 to 70.2% in 2017. This showed that the difference in the spatial distribution of China’s environmental innovation was seriously affected by the distribution of enterprises engaged in environmental innovation. Therefore, future research on China’s environmental innovation must fully consider the impact of the spatial distribution characteristics of different types of enterprises.

There are also some limitations that may exist in our study. First, we limited our data to the patent applications from the Wanfang Patent Database, which cannot avoid criticism about neglecting other data sources. Second, using only one indicator (environmental patent applications) to measure the capacity of a city’s environmental innovation is relatively weak, and building a richer evaluation system for city environmental innovation is a direction we will explore in the future. Third, although six types of environmental technologies were identified based on the work of the Organization for Economic Co-operation and Development (OECD), there are still some environmental technologies that we have not considered, such as adsorption cooling technology [52,53], advanced combustion technology, and emission reduction technology [54,55]. A broader environmental technology identification system should be constructed. Fourth, we considered the spatial relevance of urban environmental innovation but ignored the spatial diffusion of environmental innovation. In the era of innovation networking, cities participate in environmental innovation activities not only relying on their own development, but also relying on networks to obtain innovation resources. Therefore, future environmental innovation research must fully consider the externality effect brought by the innovation network.

## Figures and Tables

**Table 1:** Number of environment-related patent applications in different technical fields from 2007 to 2017 in China.

Year	Envir_M.	Energy	Green_G.	Building	Water_A.	Transp.	Total
2007	6210	32,352	843	35,850	3096	9340	87,691
2008	13,792	28,279	6885	48,444	3772	9874	111,046
2009	5895	7608	4813	33,850	3647	1099	56,912
2010	17,089	57,814	12,124	56,861	3337	22,740	169,965
2011	18,540	19,153	10,646	39,759	6301	3393	97,792
2012	25,173	34,480	13,809	62,043	5279	5221	146,005
2013	35,994	46,295	20,848	61,992	9009	11,048	185,186
2014	31,122	45,123	19,122	55,958	7088	9666	168,079
2015	41,904	93,398	28,818	128,166	9834	50,700	352,820
2016	62,345	85,142	15,247	115,247	12,471	32,415	322,867
2017	83,090	79,562	10,081	105,681	13,440	16,075	307,929

Notes: Envir_M.: environmental management technology; Green_G.: greenhouse gas treatment technology; Water_A.: water-related adaptation technology; Transp.: transportation technology.

**Table 2:** Proportion of environmental patents in overall patents from 2007 to 2017 in China.

Year	Number of Environmental Patents	Total Number of Patent Applications	Proportion of Environmental Patents
2007	87,691	407,090	21.5%
2008	111,046	469,670	23.6%
2009	56,912	575,504	9.9%
2010	169,965	711,457	23.9%
2011	97,792	957,267	10.2%
2012	146,005	1,224,727	11.9%
2013	185,186	1,382,867	13.4%
2014	168,079	1,536,629	10.9%
2015	352,820	1,910,833	18.5%
2016	322,867	2,224,683	14.5%
2017	307,929	2,696,311	11.4%

**Table 3:** Description of variables.

Variable Name	Description	Measurement
Interpreted variable		
*EIC*	Urban environmental innovation capability	The number of environment-related patent applications.
Core explanatory variable		
ER	Urban environmental regulation	The weighted average value of the treatment rates of three pollutants.
Other control variables		
U-S	Urban size	Urban registered residence population at the end of the year.
U-EDL	Urban economic development level	Urban per capita GDP.
U-TIC	Urban technological innovation capability	Urban R&D investment.
U-FDI	Urban FDI	The amount of foreign capital actually used in that year.
U-IEIC	Urban initial environmental innovation capacity	The number of environmental patents of the city in 1990.
U-CS	Urban construction	Urban fixed asset investment.
U-IS	Urban industrial structure	The proportion of secondary industry.
U-AL	Urban administrative level	1 if the city is a provincial capital, and 0 otherwise.

**Table 4:** Global Moran’s I index of urban EIC (2007, 2012, and 2017).

Year	Urban *EIC*	Different Types of Environment-Related Technologies					
		Envir_M.	Energy	Green_G.	Building	Water_A.	Transp.
2007	0.182 ***	0.193 ***	0.107 ***	0.117 ***	0.257 ***	0.111 ***	0.176 ***
2012	0.252 ***	0.201 ***	0.185 ***	0.156 ***	0.326 ***	0.178 ***	0.267 ***
2017	0.324 ***	0.330 ***	0.202 ***	0.266 ***	0.353 ***	0.267 ***	0.273 ***

Note: ***, *p* < 0.01.

**Table 5:** Top 10 cities with the most environment-related technologies (2007, 2012, and 2017).

**Year = 2007**							
**City**	**Envir_M.**	**Energy**	**Green_G.**	**Building**	**Water_A.**	**Transp.**	**Total**
Shanghai	510	3408	131	3453	356	1145	9003
Beijing	520	3589	96	3044	261	563	8073
Guangzhou	452	2758	19	2453	136	871	6689
Shenzhen	184	2489	9	2583	47	862	6174
Tianjin	248	1148	19	797	104	172	2488
Dongguan	121	745	11	894	18	405	2194
Hangzhou	152	922	32	782	110	139	2137
Ji-Nan	130	667	20	925	73	93	1908
Suzhou	120	549	36	809	47	306	1867
Ningbo	84	608	6	620	39	290	1647
**Year = 2012**							
**City**	**Envir_M.**	**Energy**	**Green_G.**	**Building**	**Water_A.**	**Transp.**	**Total**
Beijing	2669	7286	1728	4481	471	431	17,066
Shanghai	1362	2351	720	3234	414	368	8449
Guangzhou	679	2776	420	3846	172	128	8021
Suzhou	1009	1126	558	2857	207	166	5923
Hangzhou	1088	1077	922	2044	174	186	5491
Chengdu	653	824	402	1718	107	148	3852
Xi-An	423	777	292	2127	111	92	3822
Tianjin	882	667	530	1246	253	125	3703
Nanjing	746	942	649	1133	134	95	3699
Ningbo	411	495	156	1962	130	65	3219
**Year = 2017**							
**City**	**Envir_M.**	**Energy**	**Green_G.**	**Building**	**Water_A.**	**Transp.**	**Total**
Beijing	785	5020	10,731	1136	7755	797	26,224
Shenzhen	358	2479	6116	1835	8055	154	18,997
Shanghai	608	3169	4266	947	5350	461	14,801
Guangzhou	471	2640	3430	528	4414	317	11,800
Suzhou	404	3110	2399	540	3789	417	10,659
Chengdu	376	2583	2428	466	3216	302	9371
Nanjing	319	2287	2837	358	2789	303	8893
Hangzhou	337	1953	2163	334	2460	384	7631
Tianjin	344	2463	1814	314	2153	291	7379
Foshan	245	1476	920	174	4070	174	7059

Note: The underlined numbers indicate that the corresponding city ranked first in patent applications for this type of technology.

**Table 6:** Descriptive statistics of China city panel data and six types of environment-related technologies.

Variables	Obs	Mean	Std. Dev.	Min	Max
*EIC*	3014	662.066	2118.858	0	38,943
Envir_M.	3014	111.654	313.556	0	5020
Energy	3014	175.033	726.617	0	14,715
Green_G.	3014	48.516	161.997	0	2803
Building	3014	245.385	749.394	0	12,790
Water_A.	3014	25.036	65.447	0	973
Transp.	3014	56.993	230.217	0	5137
*ER*	3014	0.06	0.069	0	0.817
Ln *U-S*	3014	441.49	309.441	17.22	3375.2
Ln *U-EDL*	3014	10.19	0.736	7.782	13.056
Ln *U-TIC*	3014	9.302	1.696	−2.04	14.873
*U-FDI*	3014	69,407.898	167,333.8	0	2,113,444
*U-IEIC*	3014	22.984	58.506	0	718.5
*Ln U-CS*	3014	15.512	1.047	12.594	18.691
*U-IS*	3014	49.615	10.804	0	90.97
*U-AL*	3014	0.124	0.33	0	1

**Table 7:** Descriptive statistics for panel data of cities in three major regions of China.

**East China**					
**Variables**	**Obs**	**Mean**	**Std. Dev.**	**Min**	**Max**
*EIC*	1254	1182.068	3050.199	0	38,943
*ER*	1254	0.072	0.077	0	0.752
Ln *U-S*	1254	473.962	267.289	51.19	1442.97
Ln *U-EDL*	1254	10.426	0.701	8.476	13.056
Ln *U-TIC*	1254	9.825	1.781	4.078	14.873
*U-FDI*	1254	114,108.5	225,575.12	10	2,113,444
*U-IEIC*	1254	40.243	84.366	0	718.5
*Ln U-CS*	1254	15.834	0.999	12.971	18.571
*U-IS*	1254	49.438	8.712	0	82.28
*U-AL*	1254	0.149	0.356	0	1
**Central China**					
**Variables**	**Obs**	**Mean**	**Std. Dev.**	**Min**	**Max**
*EIC*	1188	261.213	642.137	0	6880
*ER*	1188	0.053	0.05	0.001	0.41
Ln *U-S*	1188	421.282	254.29	43.11	1244.35
Ln *U-EDL*	1188	10.079	0.677	8.232	12.456
Ln *U-TIC*	1188	9.038	1.503	4.344	13.433
*U-FDI*	1188	39,974.045	68,656.763	0	734,303
*U-IEIC*	1188	10.407	20.419	0	163.25
*Ln U-CS*	1188	15.373	0.967	12.594	18.059
*U-IS*	1188	49.416	11.651	15.17	85.92
*U-AL*	1188	0.083	0.277	0	1
**West China**					
**Variables**	**Obs**	**Mean**	**Std. Dev.**	**Min**	**Max**
*EIC*	572	354.6	1180.692	0	11,445
*ER*	572	0.05	0.081	0	0.817
Ln *U-S*	572	412.271	458.309	17.22	3375.2
Ln *U-EDL*	572	9.903	0.77	7.782	12.322
Ln *U-TIC*	572	8.705	1.553	−2.04	13.124
*U-FDI*	572	32,542.272	136,893.7	0	1,121,599
*U-IEIC*	572	11.264	21.334	0	98
*Ln U-CS*	572	15.099	1.101	12.686	18.691
*U-IS*	572	50.415	12.9	9	90.97
*U-AL*	572	0.154	0.361	0	1

**Table 8:** Regression results of the random effects model.

Variables	EIC	Envir_M.	Energy	Green_G.	Building	Water_A.	Transp.
ER^2^	896.321 ***	145.235 ***	324.754 ***	56.231 ***	101.632 ***	10.245 ***	63.247 ***
ER	−1816.837 ***	−348.393 ***	−797.105 ***	−238.132 ***	−248.698 *	−42.23 ***	−148.083 **
Ln *U-S*	0.482 ***	0.089 ***	0.108 *	0.021	0.148 **	0.029 ***	0.079 ***
Ln *U-EDL*	538.145 ***	75.462 ***	151.808 ***	9.333	200.017 ***	15.178 ***	74.9 ***
Ln *U-TIC*	14.046	19.014 ***	−5.373	7.775 ***	−1.607	2.769 ***	−5.824
*U-FDI*	0.005 ***	0.001 ***	0.001 ***	0.0004 ***	0.002 ***	0.0009 ***	0.001 ***
*U-IEIC*	18.151 ***	1.691 ***	7.765 ***	1.522 ***	5.784 ***	0.45 ***	1.022 ***
*Ln U-CS*	−187.683 ***	−28.383 ***	−59.192 ***	−1.943	−65.131 ***	−6.545 ***	−24.405 ***
*U-IS*	−22.907 ***	−4.189 ***	−6.86 ***	−0.72 ***	−7.784 ***	−0.735 ***	−1.975 ***
*U-AL*	−180.225	−48.567 ***	−117.343 **	−23.03 **	−3.391	3.827	13.869
Ln *U-S*	−1754.264 ***	−304.289 ***	−314.708	−100.657 **	−689.436 ***	−50.508 ***	−263.437 ***

Note: *** *p* < 0.01, ** *p* < 0.05, * *p* < 0.1.

**Table 9:** Regression results from different regions of China.

Variables	China	East China	Central China	West China
ER^2^	896.321 ***	103.547 ***	315.631 ***	89.243 ***
ER	−1816.837 ***	−348.393 ***	−797.105 ***	−238.132 ***
Ln *U-S*	0.482 ***	0.089 ***	0.108 *	0.021
Ln *U-EDL*	538.145 ***	75.462 ***	151.808 ***	9.333
Ln *U-TIC*	14.046	19.014 ***	−5.373	7.775 ***
*U-FDI*	0.005 ***	0.001 ***	0.001 ***	0.0008 ***
*U-IEIC*	18.151 ***	1.691 ***	7.765 ***	1.522 ***
*Ln U-CS*	−187.683 ***	−28.383 ***	−59.192 ***	−1.943
*U-IS*	−22.907 ***	−4.189 ***	−6.86 ***	−0.72 ***
*U-AL*	−180.225	−48.567 ***	−117.343 **	−23.03 **
Ln *U-S*	−1754.264 ***	−5115.433 ***	443.483	−1128.648 **

Note: *** *p* < 0.01, ** *p* < 0.05, * *p* < 0.1.

**Table 10:** Results of the spatial Durbin model (SDM).

Variables	Factors		Elimination Effect Decomposition		
	Main	W(X)	Direct Effect	Indirect Effect	Total Effect
ER^2^	864.576 ***	1124.653	1024.561 ***	554.714	746.894 ***
ER	−1717.110 ***	1242.10	−1734.926 ***	660.385	−1074.54 ***
Ln *U-S*	0.475 ***	0.00	0.483 ***	0.672	1.155
Ln *U-EDL*	437.675 ***	−51.39	448.137 ***	489.962 *	938.098 **
Ln *U-TIC*	4.26	−36.73	−5.871	−92.338	−98.21
*U-FDI*	0.005 ***	0.002 ***	0.005 ***	0.011 ***	0.016 ***
*U-IEIC*	18.288 ***	−20.246 ***	18.268 ***	−22.658 ** *	−4.39
*Ln U-CS*	−229.288 ***	97.08	−232.795 ***	−84.863	−317.658 *
*U-IS*	−16.178 ***	−4.67	−16.468 ***	−34.963 ***	−51.43 ***
*U-AL*	−26.30	−2036.330 **	−23.97	−1675.709	−1699.679
_cons	−692.31				
Spatial rho	0.587 ***				
R^2^	0.7186				
Log-likelihood	−9628.413				

Note: ***, *p* < 0.01; **, *p* < 0.05; *, *p* < 0.1.

## Data Availability

Not applicable.

## Appendix A

**Table A1:** Search strategies for the identification of environment-related technologies.

Environment-Related Technology	Description	IPC Class
Energy	Climate change mitigation technologies related to energy generation, transmission, or distribution.	F24J2, F03D9, H01L31, F03D11, H02J3, B03B13, F03D7, F03D3, F03B13, H01L51, H02N6, F03G6, F02J7, C10L5, B01J2, B09B3, F23L15, F23J15, F23L7, C10J3, F23D14, F27D17, G21C15, G21D3, G21C13, G21D1, G22C16, G06Q10, H02J13, H01B12, G06Q50, H01M2, H01M8, H01M10, H01M4, H02J7, F28D20, C25B1, G06F17, H02J15, B01D53, B01J20, C01B31
Greenhouse gases	Capture, storage, sequestration, or disposal of greenhouse gases.	B01D53, B01J20, C01B31
Transportation	Climate change mitigation technologies related to transportation.	B60L8, F02M25, B60L15, F02M21, F01N3, B60W20, F02B29, B60W10, F01N5, F01N11, F01N9, F02M27, B61D27, B61D17, B61C3, F01D5, B64D27, B64C1, B64C23, B64D11, B64C25, B63B1, B63H19, B63H13, B63H21, B60L11, H02J7, H01M8, H02J17, B60L3, B60K1
Building	Climate change mitigation technologies related to buildings.	F03D9, F24D17, H01L31, E04H1, F21S9, H05B33, F21S8, F21S2, F21V29, H05B41, F21V23, F24F5, F24H8, F24F11, F25B15, F25B29, F24F6, F24F1, F24D3, F24F12, F24H4, F24J2, F24C3, F24B1, B66B25, B66B9, B66B1, B66B11, B66B23, G08C17, G05B19, H04W52, G06F1, H04L29, F24D19, H02M3, H02M1, H02J3, H02M7, H05B37, E04B1, A01G9, E06B3, E04D13, E04D11, H02J13, H01M8, H02J9, G01D4, H02J7
Environmental management	Technologies of air pollution abatement, water pollution abatement, waste management, soil remediation, and environmental monitoring.	B01D53, F23J15, F27B1, C21B7, C21C5, F23B80, F23C9, F23C10, F02M3, B01J23, F01M13, F02D21, G01M15, F02B47, F02D41, F02D43, F02D45, F02M23, F02M25, 02M27, F02M31, F02M39, F02P5, B01D46, B01D47, B01D49, B01D50, B01D51, B03C3, F01N3, F01N5, F01N7, F01N13, F01N9, C10L10, B63J4, C02F, E03C1, E03F, E02B15, B63B35, C09K3, E01H15, B65F, A23K1, A43B1, A43B21, B03B9, B22F8, B29B7, B29B17, B30B9, B62D67, B65H73, B65D65, C03B1, C03C6, C04B7, C04B11, C04B18, C04B33, C08J11,C09K11, C10M175, C22B7, C22B19, C22B25, D01G11, D21B1, D21C5, D21H17, H01B15, H01J9, H01M6, H01M10, C05F1, C05F5, C05F7, C05F9, C05F17, C10L5, F23G5, F23G7, B09B, C10G1, A61L11, B09C, F01N11, G08B21
Water adaptation	Technologies of water conservation and availability.	F16K21, F16L55, E03C1, E03D3, E03D1, A47K11, E03D13, E03D5, E03B1, Y02B40, A01G25, C12N15, F01K23, F01D11, F17D5, G01M3, E03B5, E03B3, E03B9, E03B11
